# Pleural Tap-Guided Antimicrobial Treatment for Pneumonia with Parapneumonic Effusion or Pleural Empyema in Children: A Single-Center Cohort Study

**DOI:** 10.3390/jcm8050698

**Published:** 2019-05-16

**Authors:** Patrick M. Meyer Sauteur, Ariane Burkhard, Ueli Moehrlen, Christa Relly, Christian Kellenberger, Kerstin Ruoss, Christoph Berger

**Affiliations:** 1 Division of Infectious Diseases and Hospital Epidemiology, University Children’s Hospital Zurich, CH–8032 Zurich, Switzerland; patrick.meyer@kispi.uzh.ch (P.M.M.S.); arianeburkhard@bluewin.ch (A.B.); Christa.Relly@kispi.uzh.ch (C.R.); 2 Children’s Research Center, University Children’s Hospital Zurich, CH–8032 Zurich, Switzerland; Ueli.Moehrlen@kispi.uzh.ch (U.M.); Christian.Kellenberger@kispi.uzh.ch (C.K.); Kerstin.Ruoss@kispi.uzh.ch (K.R.); 3 Department of Pediatric Surgery, University Children’s Hospital Zurich, CH–8032 Zurich, Switzerland; 4 Division of Radiology, University Children’s Hospital Zurich, CH–8032 Zurich, Switzerland; 5 Emergency Department, University Children’s Hospital Zurich, CH–8032 Zurich, Switzerland

**Keywords:** antibiotics, community-acquired pneumonia, chest tube, drainage, fibrinolysis, parapneumonic effusion, pleural draining catheter, pleural tap, *Streptococcus pneumoniae*, thoracocentesis, thoracotomy, video-assisted thoracic surgery

## Abstract

Parapneumonic effusion or pleural empyema (PPE/PE) is a frequent complication of community-acquired pneumonia (CAP) in children. Different management approaches exist for this condition. We evaluated a 14-day treatment with amoxicillin (AMX) with/without clavulanic acid (AMC) confirmed or modified by microbiological findings from pleural tap. Children ≤16 years of age with radiologically diagnosed PPE/PE and initial diagnostic pleural tap were included at University Children’s Hospital Zurich from 2001–2015. AMX/AMC was given for 14 days and rationalized according to microbiological pleural tap results. Clinical and radiological follow-up was scheduled until six months or full recovery. In 114 of 147 (78%) children with PPE/PE a pathogen was identified by culture, polymerase chain reaction (PCR), and/or antigen testing. *Streptococcus pneumoniae* was detected in 90 (79%), *S. pyogenes* in 13 (11%), and *Staphylococcus aureus* in seven cases (6%), all but two cultured pathogens (96%) were sensitive to AMX/AMC. One-hundred two of 147 (69%) patients received treatment with AMX/AMC for 14 days. They recovered more rapidly than patients with a different management (*p* = 0.026). Of 139 children with follow-up, 134 (96%) patients fully recovered. In conclusion, 14-day AMX/AMC treatment confirmed and rarely modified by microbiological findings from pleural tap resulted in full recovery in >95% of children with PPE/PE.

## 1. Introduction

Parapneumonic effusion or pleural empyema (PPE/PE) develop in about 40% of children with community-acquired pneumonia (CAP) requiring hospitalization [1]. The incidence of PPE/PE in children has increased worldwide in recent decades (5.5 per 100,000 in the U.S. in 2006) [2,3,4]. PPE/PE is clinically suspected in children with CAP that show persisting fever and lack of improvement 48–72 h after initiation of antibiotic treatment [5]. Sampling of pleural fluid in PPE/PE patients by simple pleural tap provides both diagnostic and therapeutic benefit [5]. Microbiologic workup from pleural fluid or blood in children most frequently reveals *Streptococcus pneumoniae* as the etiologic agent, even after the introduction of the pneumococcal conjugate vaccines (PCVs) [2,3,4,6,7]. The dynamic evolution of the inflammatory process in PPE/PE is divided into three stages, characterized as follows: stage 1, free-floating fluid, stage 2, fluid loculated by fibrous septations, and stage 3, consolidation (empyema) [8]. This staging and the size of the effusion influence the management of PPE/PE [5]. Yet, the optimal management of PPE/PE is controversial [9]. The applied therapeutic strategies include administration of antibiotics alone (i.e., amoxicillin (AMX) with/without clavulanic acid (AMC)) or combined with interventions, such as pleural tap, pleural draining catheter, fibrinolysis, video-assisted thoracoscopic surgery (VATS), or thoracotomy [1,5,10,11]. Currently, not only the indication but also the impact of these interventions on duration of hospitalization and outcome are controversially discussed [1,9,10,11,12,13], and its clinical application often follows rather in-house experience-based protocols than evidence.

Here, we present a 15-year single-center cohort study of children with PPE/PE by implementing a management algorithm including diagnostic pleural tap and a 14-day course of empirical antibiotic treatment. We hypothesized that these patients recover following a 14-day course of AMX/AMC treatment confirmed or modified by microbiological findings from pleural tap. We further compared the outcome for PPE/PE patients managed without surgical interventions (i.e., only diagnostic pleural tap) or with surgical interventions (e.g., pleural draining catheter and VATS).

## 2. Experimental Section

### 2.1. Ethics Statement

Ethical approval for this study was granted by the ethics committee of Canton Zurich, Switzerland (protocol no. 2016-01331; approved: 20 September 2016).

### 2.2. Patients

Children ≤16 years of age with radiologically confirmed PPE/PE that underwent a diagnostic pleural tap at University Children’s Hospital Zurich between January 2001 and December 2015 were included in this retrospective analysis. Excluded were children with underlying diseases and immunosuppression. Patients were treated according to a defined management algorithm including empirical antibiotic treatment given for 14 days and rationalized according to microbiological findings from initial pleural tap. The 14-day course of antibiotic treatment was irrespective of pretreatment duration and regimen. Indications for surgical interventions included high grade or continuing respiratory compromise, seropneumothorax, and/or large effusions with mediastinal shift [5]. Surgical interventions were defined as invasive procedures such as chest tube drainage ± intrapleural fibrinolytic therapy, VATS, and thoracotomy. The choice of the surgical intervention was made interdisciplinary by responsible surgeons and pediatricians. Clinical and radiological follow-up was scheduled up to 6 months or until full recovery.

### 2.3. Data Collection

Clinical and radiological data were collected at inclusion and at 1-, 3-, and/or 6-month follow-ups. Biological samples (blood and pleural fluid) were collected at inclusion.

### 2.4. Imaging

Images were judged by a radiologist during routine radiological assessment. These findings and the corresponding images were reviewed by two of the authors, who were blinded to details of patient histories. The images were graded (radiograph) and classified (ultrasound) as follows:

#### 2.4.1. Quantification (Grade 1–3)

The size of effusion in chest radiograph was assessed according to Bradley et al. [5]: grade 1, small (opacifies less than one-fourth of hemithorax); grade 2, moderate (opacifies less than half of the hemithorax); and grade 3, large (opacifies more than half of the hemithorax).

#### 2.4.2. Classification (Stage 1–3)

Sonographic staging was performed according to Kim et al. [8]: stage 1, exudative (free-floating fluid without loculations or consolidations); stage 2, fibrino-purulent (fluid loculated by fibrous septations); and stage 3, organized (echogenic, solid-appearing pleural plaque of >1/3 of PPE/PE with or without some loculation of fluid).

### 2.5. Microbiological Analyses

#### 2.5.1. Pleural Fluid

Gram stain and conventional bacterial cultures for aerobic an anaerobic organisms were performed on all pleural fluid specimens [14]. Antigen testing or nucleic acid amplification through polymerase chain reaction (PCR) was primarily used on culture-negative pleural fluid specimens to increase the detection of pathogens in pleural fluid. Antigen testing for *S. pneumoniae* and *S. pyogenes* was performed with commercially available tests (Pneumoslide test (BD Diagnostics, Sparks Glencoe, MD, USA) and Pastorex Strep (Bio-Rad Laboratories Inc., Marne-La-Coquette, France)). Specific PCRs for *S. pneumoniae* and pneumococcal serotypes, as well as the broad-range PCR were used as previously described [15,16,17].

#### 2.5.2. Blood Culture

Bacterial blood cultures for aerobic and anaerobic organism were performed on all patients with PPE/PE as described previously [17].

### 2.6. Follow-up and Outcome

Outcome parameters were length of hospital stay (LOS) and complete clinical and radiological recovery (i.e., good outcome). LOS was defined as time between first radiological proof of PPE/PE (time point of inclusion, day 0) and day of hospital discharge. Poor outcome was defined as no clinical recovery and/or abnormal chest radiograph according to the British Thoracic Society (BTS) guidelines [1,11].

### 2.7. Statistical Analysis

Categorical variables were compared by using χ^2^ or Fisher’s exact analyses. Continuous variables were compared by using Wilcoxon rank-sum (Mann-Whitney *U*) analysis. The log-rank test was used to compare the time to outcome. All reported *p* values are 2-tailed with significance at <0.05. Analyses were performed using the R software environment (version 3.4.0, R Core Team, Vienna, Austria) [18].

## 3. Results

**Figure 1:**
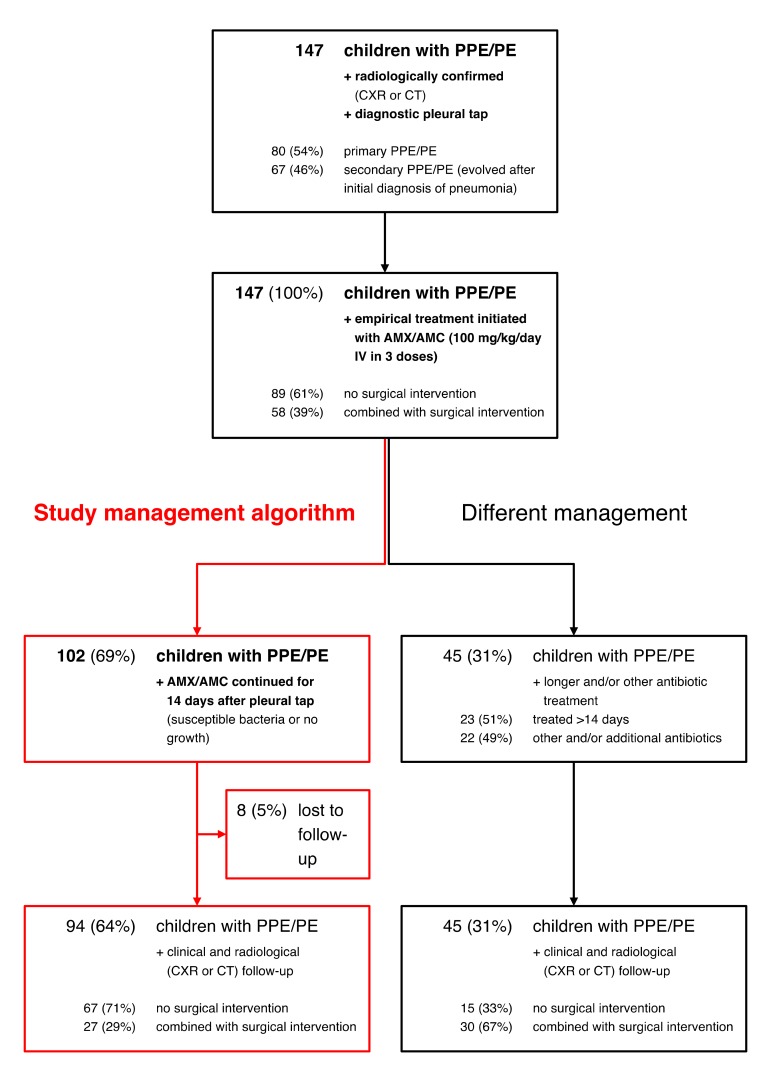
Study profile. AMC, amoxicillin-clavulanate; AMX, amoxicillin; PE, pleural empyema; PPE, parapneumonic effusion; CT, computed tomography; CXR, chest radiograph.

During the 15-year study period, 147 children with radiologically confirmed PPE/PE who underwent a diagnostic pleural tap were included (Figure 1). Numbers of PPE/PE cases within 5-year-intervals were 24 (16%), 60 (41%), and 63 (43%) from 2001–2005, 2006–2010, and 2011–2015, respectively. Seventy-three (50%) were male and the median age was four years (interquartile range (IQR) 3–6 years). At admission, 80 (54%) children presented with PPE/PE (primary PPE/PE) and 67 children (46%) with CAP and a subsequent pleural effusion (secondary PPE/PE). The median time between onset of initial symptoms and diagnosis of PPE/PE was five days (IQR 3–9 days) for children with primary PPE/PE and nine days (IQR 7–11 days) for children with secondary PPE/PE (*p* < 0.001). Baseline characteristics of included PPE/PE patients are shown in Table 1, including comparison between patients managed with pleural tap alone and with surgical interventions.

A pathogen was identified in 114 out of 147 (78%) children with PPE/PE and initial pleural tap (Table 2). Pathogens were detected by conventional bacterial cultures of pleural fluid and/or blood in 48 (33%) patients. Antimicrobial treatment >24h prior to the diagnosis of PPE/PE was administered in 110 (75%) patients. The median treatment duration before sample collection was three days (IQR 1–5 days). Negative pleural fluid cultures were more frequently observed when samples were collected >24 h after initiation of antimicrobial treatment compared to those collected earlier (83% vs. 22%, *p* < 0.001). Among patients with negative pleural fluid cultures (*n* = 99, 67%), PCR and antigen testing allowed to identify a pathogen in two thirds of children (*n* = 66/99, 67%).

*S. pneumoniae* was detected in 90 (79%), *S. pyogenes* in 13 (11%) and *Staphylococcus aureus* in seven (6%) cases. Other bacteria included non-typeable *Haemophilus influenzae*, *Streptococcus intermedius*, *Actinomyces* spp., and *Fusobacterium necrophorum*. *S. pneumoniae* serotypes could be identified in 23 of the 26 (88%) cultured bacterial isolates. The most common serotypes were serotype 3 (*n* = 7/23, 30%) and serotype 19A (*n* = 5/23, 22%; Table 2). Infections with vaccine serotypes despite vaccination with PCVs were observed in seven patients (serotype 3, *n* = 4; serotype 19A, 9V, and 1, *n* = 1).

Empirical treatment with AMX/AMC was initiated in all children at inclusion. AMX/AMC was continued if pleural fluid and/or blood culture isolates were susceptible to this treatment (*n* = 46), *S. pneumoniae* or *S. pyogenes* were identified by PCR and/or antigen testing (*n* = 66), or if no pathogen was identified by bacterial cultures, PCR, or antigen testing (*n* = 33). All but two (*n* = 46/48, 96%) culture isolates were susceptible to AMX/AMC. Resistant pathogens included penicillin-resistant *S. pneumoniae* (*n* = 1) and methicillin-resistant *S. aureus* (MRSA, *n* = 1), both treated with vancomycin based on antibiotic susceptibility testing. According to the management algorithm, 102 (69%) children with PPE/PE received AMX/AMC treatment for a total duration of 14 days (Figure 1). Not managed according to the management algorithm were PPE/PE patients receiving another antibiotic regimen because of sepsis or adverse drug reactions (*n* = 22), or with a treatment duration >14 days because of nosocomial infections apart from PPE/PE, including catheter-related bloodstream infections or skin and soft tissue infections (*n* = 23).

Surgical interventions were performed in 58 (39%) children with PPE/PE, including chest tube drainage alone (*n* = 36) or with intrapleural fibrinolytic therapy (*n* = 1), VATS (*n* = 16), and thoracotomy (*n* = 5). Thereof, 27 (47%) children received a secondary intervention later than three days after diagnosis of PPE/PE. Compared to PPE/PE patients without surgical intervention, patients that underwent surgical intervention were more likely to have a positive pleural fluid Gram stain (*p* = 0.002), an increased white blood cell (WBC) count (*p* < 0.001), and a large size (*p* < 0.001) and organized (*p* = 0.011) effusion (Table 1). The median LOS of all 147 children with PPE/PE was 11 days (IQR 8–19 days). PPE/PE patients with surgical interventions were significantly longer hospitalized than those with pleural tap alone (LOS 21 vs. 9 days, *p* < 0.001; Table 3).

**Figure 2:**
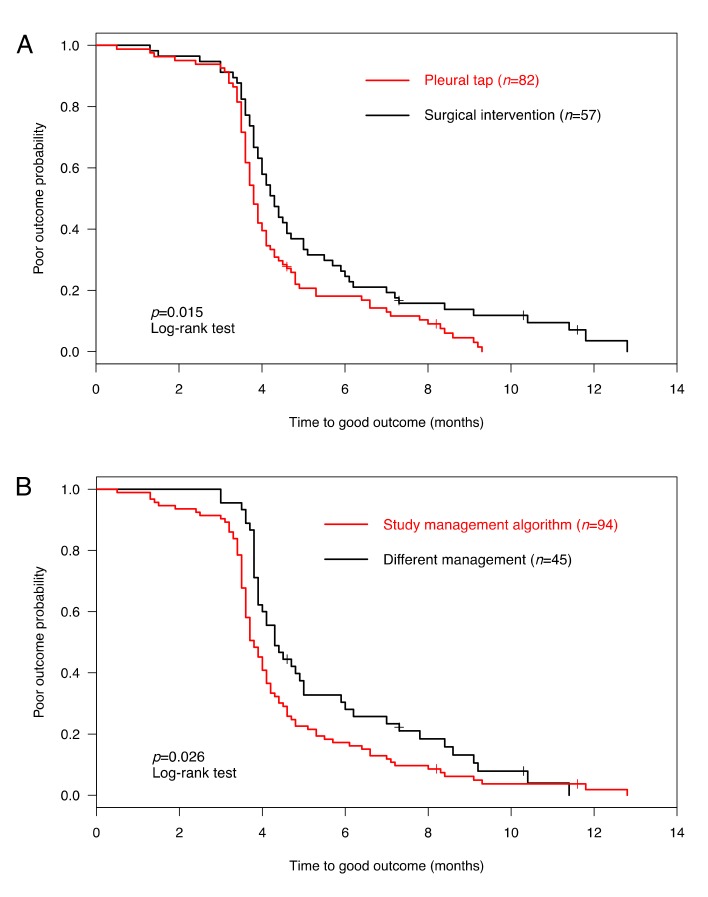
Outcome analysis of 139 children with parapneumonic effusion or pleural empyema (PPE/PE). (**A**) Time to good outcome of PPE/PE with pleural tap alone (red) or surgical intervention (black) (i.e., pleural draining catheter ± fibrinolysis, video-assisted thoracoscopic surgery, or thoracotomy). (**B**) Time to good outcome of PPE/PE managed according to the study management algorithm (red) or with different management (black). Poor outcome was defined as no clinical recovery and/or abnormal chest radiograph according to the British Thoracic Society (BTS) guidelines [1]. The log-rank test was used to compare the time to outcome.

Among 139 (95%) children with follow-up, complete clinical and radiological recovery occurred in 134 (96%) patients. Thereof, 51% and 79% fully recovered until four and six months, respectively. The median time to complete recovery was 4.0 months (range 0.5–12.8 months). Although the final outcome was comparable between patients with surgical interventions and those with pleural tap alone (complete recovery in 95% vs. 98%, *p* = 0.401), patients that underwent surgical intervention needed significantly more time to recover (Figure 2A). Our final aim was to assess the outcome of a management algorithm including diagnostic pleural tap and a 14-day course of AMX/AMC. Two (2%) PPE/PE patients managed with the management algorithm and three (5%) with different management experienced a poor outcome (*p* = 0.401). In fact, PPE/PE patients managed according to the management algorithm recovered more rapidly than patients with a different management (Figure 2B). The characteristics of the five (4%) PPE/PE patients with poor outcome, defined as incomplete clinical and/or radiological recovery, are shown in Table 4. Thereof, only two (1%) suffered from clinical impairment (chronic pneumopathy), while the three others reached complete clinical recovery.

## 4. Discussion

In this 15-year single-center cohort study in Switzerland, we showed that an initial tap-guided management for children with PPE/PE including treatment with AMX/AMC for 14 days from pleural tap resulted in full recovery in >95% of the cases. PPE/PE is a frequent complication of CAP for which wide variations in management approaches exist [1,2,3,4,12,19,20,21,22,23]. We retrospectively analyzed the feasibility and outcome following an uniform management algorithm for childhood PPE/PE that included (I) enforced pathogen identification with initial diagnostic pleural tap, (II) 14-day-course of antibiotics, i.e., AMX/AMC or targeted treatment according to microbiological test results, and (III) surgical interventions restricted to cases with respiratory compromise, seropneumothorax, and/or large effusions with mediastinal shift. Using this management algorithm, we corroborate previous findings on the significantly increased percentage of pathogen identification in pleural fluid by extended diagnostic techniques compared to the yield from blood cultures alone [13,24,25,26,27,28,29]. In our study, initial pleural tap increased pathogen detection from 17% detection rate by blood cultures to 78% detection rate by microbiological analyses of pleural fluid of PPE/PE patients. PCR and antigen testing enabled pathogen detection in almost half of all patients that were already treated with antibiotics. Pleural tap is a minimally invasive procedure with very rare complications [30] and sonographic guidance can facilitate the procedure [23]. Our data support current guidelines in recommending a simple pleural tap for children with PPE/PE, as it provides both diagnostic and therapeutic benefit [5,11]. Pleural tap is a prerequisite for the narrow-spectrum antibiotic regimen of limited duration as suggested by our management algorithm.

*S. pneumoniae* was the most frequently identified pathogen, followed by *S. pyogenes* and *S. aureus*, which is in agreement with previously published data on the etiology of PPE/PE in children worldwide [26,28,31,32,33,34,35,36]. The study period covered the introduction of PCVs in Switzerland among all children <2 years of age (with a catch-up dose up to five years of age) with PCV7 in 2006 and a switch to PCV13 in 2011 [17]. Infections with vaccine serotypes despite vaccination with PCVs occurred in seven patients, mainly serotype 3, which is included in PCV13 [17]. Data on PCV coverage status in our study is limited, and we thus cannot assess the influence of PCVs on pneumococcal PPE/PE in this setting.

Our data confirm that empirical antibiotic treatment must target *S. pneumoniae* and cover *S. pyogenes* and *S. aureus* [11,28,35]. The choice of the empirical antibiotic agent should be guided by local resistance data and the emergence of resistant organisms [11]. AMX/AMC is one recommended suitable option for PPE/PE following CAP according to current guidelines [1,5,11,37]. AMC can be narrowed to AMX in case of *S. pneumoniae* and *S. pyogenes* detection [5]. In fact, 46 out of 48 (96%) cultured pathogens in this study were susceptible to treatment with AMX/AMC. Notably, pleural tap allowed also the detection of *Actinomyces* spp. in pleural fluid culture of one patient, which required AMC treatment for a prolonged duration of six months. The two (4%) AMX/AMC-resistant pathogens isolated from pleural fluid were penicillin-resistant *S. pneumoniae* and MRSA, respectively. However, the prevalence of penicillin-resistant *S. pneumoniae* [38] and MRSA [39] is still very low in Switzerland and may not justify an antibiotic escalation regimen for the empirical treatment of PPE/PE. Even less if the management includes an initial diagnostic pleural tap to identify pathogens that are resistant against the empirical treatment. The pleural tap-guided management will allow correct and targeted antibiotic treatment of limited duration that prevents further emergence of resistant pathogens.

No evidence exists about the duration of antibiotic treatment for PPE/PE [11,37,40]. Intravenous antibiotic treatment is usually continued until there is obvious clinical improvement, and subsequent oral antibiotic treatment such as AMX/AMC is administered for 1–4 weeks following discharge [37,40]. In this study, we showed that treatment with AMX/AMC restricted to total 14 days following pleural tap led to good outcome in the vast majority of cases (98%), independent of a management with tap-guided antibiotic treatment alone or with surgical intervention. Notably, PPE/PE patients show variable disease courses with sometimes very protracted recovery due to pronounced inflammatory processes within the pleural cavity, even after successful killing of organisms at the local site. This may have led to the varying recommendations for extensive treatment durations in children with PPE/PE [11,13,40]. However, the median length of treatment in our study was 14 days for all antibiotic regimens and irrespective of interventions, and we thus propose to limit treatment duration for this condition to a total of two weeks following diagnosis.

The indication for and role of surgical interventions in the management of childhood PPE/PE is controversial [20,37,41]. Because fibrin formation impairs drainage of pleural fluid, surgical interventions are recommended for PPE/PE patients in which pleural fluid has already progressed to an effusion with multiple loculations [10]. It has been suggested that pleural draining catheter with fibrinolysis may be a preferred primary therapy in empyema and that VATS should be reserved for failure of initial management with antibiotics alone [37]. However, recent studies reported a successful management without surgical intervention in a considerable number of PPE/PE patients even with effusions loculated by fibrous septations and consolidations [9,41]. In line with these data, our study demonstrates that PPE/PE could be managed with a simple diagnostic pleural tap without surgical interventions in 61% of all cases, half of which had a large size of effusion. Surgical interventions were restricted to cases with a high degree of respiratory compromise, seropneumothorax, and/or large effusions causing mediastinal shift. There was no difference in outcome of children with different methods of surgical interventions (data not shown). The overall median LOS in our study with 11 days was significantly shorter compared to other European studies (Germany, 17 days [12]; Poland, 20 days [36]; Spain, 17 days [42]; and Israel, 15 days [43]). In contrast to other studies [12,44], our PPE/PE patients with surgical interventions were significantly longer hospitalized than those with pleural tap alone, and required significantly longer time to full recovery. Overall, the full recovery in 96% of children with PPE/PE further suggests that the presented management algorithm may be indeed a safe and minimal-invasive approach to manage PPE/PE in children with a rational and targeted antibiotic regimen following pleural tap.

The strengths of this study are the long observational period and the single-center design, which enabled us to implement and standardize a uniform management algorithm and closely follow a well-defined study cohort. However, the study also has several limitations. First, our center is a large tertiary university hospital and many patients were already pretreated and secondarily referred by primary physicians or secondary centers. Second, due to the retrospective study design we are unable to provide information on total numbers of cases with CAP, PPE/PE following CAP who did not undergo a pleural tap procedure, and PPE/PE in children with underlying diseases or immunosuppression. Third, as previously mentioned [3], surgical interventions are reflected by the current skills and experience of treating surgeons and pediatricians. However, apart from performing VATS instead of thoracotomy because of several evidence-based advantages [20,37], no major intervention changes were introduced during the study period. Finally, although PCR and antigen testing substantially enabled increased pathogen detection, pleural fluid was not in all cases investigated by both PCR and antigen testing techniques.

## 5. Conclusions

The outcomes of this study strongly suggest that a standardized management of PPE/PE in children, including an initial diagnostic pleural tap and consistent prescription of a two-week-course of antibiotic treatment adjusted to microbiological test results, is effective and safe in most children. This finding is consistent with studies that have been done previously. The empirical treatment regimen including monotherapy with AMX/AMC can be adapted in countries with low prevalence of penicillin-resistant *S. pneumoniae* and MRSA, and AMC can be narrowed to AMX in case of *S. pneumoniae* and *S. pyogenes* detection in this setting. Surgical interventions may be restricted to cases with a high degree of respiratory compromise, seropneumothorax, and/or large effusions causing mediastinal shift. Although children with PPE/PE show variable disease courses with protracted recovery the overall prognosis of pediatric PPE/PE is good. Our pleural tap-guided treatment resulted in full recovery in over 95% of children with PPE/PE, irrespective of the management with pleural tap alone or surgical interventions. A prospective multicenter study is needed in the future to verify these single center findings within the pediatric population.

## Figures and Tables

**Table 1:** Baseline characteristics of the 147 included children with parapneumonic effusion or pleural empyema (PPE/PE).

Baseline Characteristics	Total Number of PPE/PE Patients	PPE/PE Patients Without Surgical Intervention	PPE/PE Patients with Surgical Intervention	OR (95% CI)	*p* Value
	(*n* = 147)	(*n* = 89, 61%)	(*n* = 58, 39%)		
**Demographic characteristics**					
Sex (male), *n* (%)	73 (50)	48 (54)	25 (43)	1.5 (0.8–3.2)	0.238
Age (years), median (IQR)	4 (3–6)	4 (3–7)	4 (2–5)		0.098
**Clinical characteristics**					
Duration of prodromal fever and/or respiratory symptoms (days), median (IQR)	7 (4–10)	7 (5–11)	6 (4–9)		0.060
Diagnosis of pneumonia prior to diagnosis of PPE/PE, *n* (%)	67 (46)	49 (55)	18 (31)	2.7 (1.3–5.8)	0.007
Antibiotics prior to diagnosis of PPE/PE, *n* (%)	110 (75)	72 (81)	38 (66)	2.2 (1.0–5.1)	0.051
Duration from start antibiotics to diagnosis of PPE/PE (days), median (IQR)	3 (1–5)	4 (2–6)	2 (0–4)		<0.001
Symptoms at diagnosis of PPE/PE:					
• Chest pain, *n* (%)	29 (20)	21 (24)	8 (14)	1.9 (0.7–5.4)	0.212
• Sepsis, *n* (%)	32 (22)	10 (11)	22 (38)	0.2 (0.1–0.5)	<0.001
Inflammatory parameters:					
• WBC count (G/L), median (IQR)	15.1 (8.6–22.4)	15.8 (10.0–21.2)	13.6 (8.1–22.9)		0.636
• CRP (mg/L), median (IQR)	200 (157–317)	195 (152–314)	202 (160–318)		0.370
**Pleural effusion characteristics**					
Pleural fluid:					
• Gram stain positive, *n* (%)	32/125 (26)	14/81 (17)	18/44 (41)	0.3 (0.1–0.7)	0.002
• WBC count (cells/μl), median (IQR)	6604 (1899–69,112)	4258 (1650–10,964)	71,000 (5900–121,980)		<0.001
Size of effusion [5]:					
• Grade 1 (small, <¼ hemithorax), *n* (%)	17/142 (12)	13/86 (15)	4/56 (7)	2.3 (0.7–10.2)	0.194
• Grade 2 (moderate, ¼–½ hemithorax), *n* (%)	35/142 (25)	30/86 (35)	5/56 (9)	5.4 (1.9–19.2)	<0.001
• Grade 3 (large, >½ hemithorax), *n* (%)	90/142 (63)	43/86 (50)	47/56 (84)	0.2 (0.1–0.5)	<0.001
Sonographic staging [8]:					
• Stage 1 (exudative), *n* (%)	48/115 (42)	34/75 (45)	14/40 (35)	1.5 (0.7–3.7)	0.325
• Stage 2 (fibrino-purulent), *n* (%)	54/115 (47)	37/75 (49)	17/40 (43)	1.3 (0.6–3.1)	0.558
• Stage 3 (organized), *n* (%)	13/115 (11)	4/75 (5)	9/40 (23)	0.2 (0.1–0.8)	0.011

CRP, C-reactive protein; IQR, interquartile range; PE, pleural empyema; PPE, parapneumonic effusion; WBC, white blood cell. Differences between PPE/PE patients without and with surgical intervention were determined by the Mann-Whitney *U* test (medians) and Fisher’s exact test (proportions).

**Table 2:** Microbiological diagnosis in 114 out of the 147 children with PPE/PE.

Pathogen Detection (*n* = 114, 78%)	Total	Pleural Fluid	PCR (*n*)	Antigen (*n*)	Blood
	*n* (%)	Culture (*n*)			Culture (*n*)
***Streptococcus pneumoniae***	90 (79)	13 ^a^	39	84 ^b^	16 ^a^
Vaccine serotypes ^c^:					
1	4	2	2	4	2
3	7	3	1	4	5
7F	3	1		3	3
9V	1	1		1	
19A	5	3	1	4	3
14	3			3	3
NA	3	3		2	
***Streptococcus pyogenes***	13 (11)	12 ^d^	2 ^e^	1	4 ^d^
***Staphylococcus aureus***	7 (6)	6			4
MSSA	6	5 ^f^			4 ^f^
MRSA	1	1			0
**Others ^g^**	4 (4)	3 ^h^	1		1
**Total**	114	34	42	85	25

MRSA, methicillin-resistant *S. aureus*; MSSA, methicillin-sensitive *S. aureus*; PCR, polymerase chain reaction. ^a^ *n* = 3 with pleural fluid culture and blood culture positive; ^b^ *n* = 12 with antigen and pleural fluid culture positive, *n* = 38 with antigen and PCR positive; ^c^ PCV7 (Prevenar 7^®^, introduced in 2006): serotypes 4, 6B, 9V, 14, 18C, 19F, 23F; PCV13 (Prevenar 13^®^, introduced in 2011): serotypes 1, 3, 4, 5, 6A, 6B, 7F, 9V, 14, 18C, 19A, 19F, 23F; ^d^ *n* = 4 with pleural fluid culture and blood culture positive; ^e^ *n* = 1 with PCR and antigen positive, *n* = 1 with PCR and pleural fluid culture positive; ^f^ *n* = 3 with pleural fluid culture and blood culture positive; ^g^ non-typeable *Haemophilus influenzae* (*n* = 1, pleural fluid culture and blood culture positive), *Streptococcus intermedius* (*n* = 2, pleural fluid culture positive), *Actinomyces* spp. (*n* = 1, pleural fluid culture positive), and *Fusobacterum necrophorum* (*n* = 1, detected by broad-range PCR); ^h^ *n* = 1 pleural fluid positive for both *Actinomyces* spp. and *S. intermedius*. All cultured isolates (*n* = 48) were tested for antimicrobial susceptibility according to European Committee on Antimicrobial Susceptibility Testing (EUCAST) [14]. *S. pyogenes* and *S. pneumoniae* isolates were susceptible to penicillin (minimum inhibitory concentration (MIC) for *S. pneumoniae* <0.06 mg/L, determined by Etest (AB Biodisk, Solna, Sweden)) in 100% (*n* = 12/12) and 96% (*n* = 25/26), respectively. *S. aureus* isolates were susceptible to cefoxitin (disk diameter ≥22 mm, determined by cefoxitin disk diffusion test) in 86% (*n* = 6/7). Children with these susceptible isolates (*n* = 43) were treated with amoxicillin (AMX) with/without clavulanic acid (AMC), and the isolates were not systematically tested against other antibiotics over the 15-year study period. This was also true for children with other bacterial isolates (*n* = 3) that were all treated with AMX/AMC according to antimicrobial susceptibility testing. One *S. pneumoniae* isolate was resistant against penicillin (MIC > 2 mg/L) and one MRSA was detected (cefoxitin disk diameter <22 mm; in addition, detection of penicillin binding protein 2a (PBP2a) direct from *S. aureus* culture isolate by the immunochromatographic assay Alere PBP2a Culture Colony Test (Alere, Scarborough, ME, USA)). Their susceptibility testing results against other antibiotics were as follows: *S. pneumoniae*, clindamycin susceptible (S), erythromycin S, vancomycin S; MRSA, clindamycin S, gentamicin S, vancomycin S, meropenem resistant (R).

**Table 3:** Management and outcome of the 147 children with PPE/PE.

Management	Total Number of PPE/PE Patients (*n* = 147)	PPE/PE Patients Without Surgical Intervention (*n* = 89, 61%)	PPE/PE Patients with Surgical Intervention (*n* = 58, 39%)	OR (95% CI)	*p* Value
**Hospitalization**					
LOS (days), median (IQR)	11 (8–19)	9 (6–13)	21 (12–34)		<0.001
**Antibiotic treatment**					
Duration (d) after time point of diagnosis, median (range)	14 (2–255)	14 (4–20)	14 (2–255)		0.016
Total duration (d) including pretreatment, median (range)	16 (13–255)	16 (14–30)	16 (13–255)		0.750
**Outcome**	**(*n* = 139)**	**(*n* = 82, 59%)**	**(*n* = 57, 41%)**		
Complete recovery, *n* (%)	134 (96)	80 (98)	54 (95)	2.2 (0.2–27.3)	0.401
Time to complete recovery (months), median (IQR)	4.0 (3.6–5.1)	3.8 (3.5–4.7)	4.3 (3.7–6.0)		0.023
Complete recovery achieved in:					
• ≤4 months FUP, *n* (%)	71 (51)	48 (59)	23 (41)	2.1 (1.0–4.4)	0.040
• 4–6 months FUP, *n* (%)	39 (28)	19 (23)	20 (35)	0.6 (0.3–1.3)	0.131
• >6 months FUP, *n* (%)	24 (17)	13 (16)	11 (19)	0.8 (0.3–2.1)	0.652

AMC, amoxicillin-clavulanate; AMX, amoxicillin; FUP, follow-up; IQR, interquartile range; LOS, length of hospital stay; PE, pleural empyema; PPE, parapneumonic effusion. Complete recovery is defined as clinical and radiological recovery. Differences between PPE/PE patients without and with surgical intervention were determined by the Mann–Whitney *U* test (medians) and Fisher’s exact test (proportions).

**Table 4:** Complications in children with PPE/PE.

Patient	1	2	3	4	5
Diagnosis	PPE/PE	PPE/PE with ARDS and DIC	Necrotizing PPE/PE with MOF	PPE/PE	PPE/PE
**Demographic characteristics**					
Sex	Female	Female	Male	Male	Male
Age (y)	1	12	3	2	8
Previous medical history				Recurrent viral bronchitis, trisomy 21	Recurrent viral bronchitis
**Microbiology**					
Etiology	***S. pneumoniae*** (serotype unknown)	**MSSA**	**MRSA**	***S. pneumoniae*** (serotype 7F)	***S. pneumoniae*** (serotype 1)
Diagnostic test	Antigen test (pleural fluid)	Culture (blood)	Culture (pleural fluid)	Culture (blood, pleural fluid)	Culture (blood), PCR and antigen test (pleural fluid)
Other detected pathogens	NA	Influenza B (NPS)	Candida (CRBI)	Influenza A (NPS)	NA
**Pleural effusion**					
Grading [5]	3 (large)	2 (moderate)	3 (large)	3 (large)	3 (large)
Staging [8]	NA	NA	1 (exudative)	2 (fibrino-purulent)	1 (exudative)
**Hospitalization and management**					
LOS (days)	10	39	77	19	20
ICU (days)		15	55	5	
Surgical intervention	Pleural draining catheter	Pleural draining catheter	Thoracotomy and decortication following pleural draining catheter, VA-ECMO (9 days)		
Complication	Pneumothorax, bronchopleural fistula	Pneumothorax, ARDS, DIC	Bronchopleural fistula, pneumothorax, necrotizing pneumonia, MOF, ischemic cerebral lesions, CRBI		
Antibiotics (total duration in days)	AMC (14)	AMX, gentamicin, flucloxacillin, clindamycin (21)	AMC, teicoplanin, gentamicin, meropenem, vancomycin, linezolid (28)	AMC (14)	AMX (18)
**FUP and outcome**					
Last FUP (months)	12	7	10	8	5
Clinical recovery	+	–	+	–	+
Details	NA	Reduced lung function and exercise capacity	NA	Reduced lung function and exercise capacity	NA
Radiological recovery	–	–	–	–	–
Details	Persistent bulla, residual pleural thickening	Areas of air trapping	Residual pleural thickening	Emphysema, atelectasis	Residual pleural thickening

AMC, amoxicillin-clavulanate; AMX, amoxicillin; ARDS, acute respiratory distress syndrome; CRBI, catheter-related bloodstream infection; DIC, disseminated intravascular coagulation; FUP, follow-up; ICU, intensive care unit; LOS, length of hospital stay; MOF, multiple organ failure; MRSA, methicillin-resistant *S. aureus*; MSSA, methicillin-sensitive *S. aureus*; NA, not available; NPS, nasopharyngeal secretions; PE, pleural empyema; PPE, parapneumonic effusion; VA-ECMO, veno-arterial extracorporeal membrane oxygenation.
